# Effects of Lycopene Attenuating Injuries in Ischemia and Reperfusion

**DOI:** 10.1155/2022/9309327

**Published:** 2022-10-07

**Authors:** Sijia Wu, Xiajun Guo, Jia Shang, Yuanyuan Li, Wanglin Dong, Qianwen Peng, Zhenxing Xie, Chaoran Chen

**Affiliations:** ^1^Institute of Nursing and Health, College of Nursing and Health, Henan University, Kaifeng, Henan, China; ^2^Arts Department, School of Kaifeng Culture and Tourism, Kaifeng, Henan, China; ^3^School of Basic Medical Sciences, Henan University, Jinming Avenue, Kaifeng, Henan 475004, China

## Abstract

Tissue and organ ischemia can lead to cell trauma, tissue necrosis, irreversible damage, and death. While intended to reverse ischemia, reperfusion can further aggravate an ischemic injury (ischemia-reperfusion injury, I/R injury) through a range of pathologic processes. An I/R injury to one organ can also harm other organs, leading to systemic multiorgan failure. A type of carotenoid, lycopene, has been shown to treat and prevent many diseases (e.g., rheumatoid arthritis, cancer, diabetes, osteoporosis, male infertility, neurodegenerative diseases, and cardiovascular disease), making it a hot research topic in health care. Some recent researches have suggested that lycopene can evidently ameliorate ischemic and I/R injuries to many organs, but few clinical studies are available. Therefore, it is essential to review the effects of lycopene on ischemic and I/R injuries to different organs, which may help further research into its potential clinical applications.

## 1. Introduction

Ischemia refers to insufficient blood supply to the tissues or organs of the body [1]. It is usually caused by events such as thromboembolism, trauma, atherosclerosis, tachycardia, and organ transplantation [2]. Ischemia can lead to the lack of oxygen and nutritional elements required for cell metabolism and insufficient clearance of metabolic waste [3, 4]. The direct outcomes of ischemia are cell damage, tissue necrosis, irreversible pathologies, and death [5]. The severity of ischemic damage rests with the degree and duration of blood flow restriction. In cases of the short-term or mild ischemia, a new balance formed between energy supply and utilization results in less severe injuries and blood supply can be recovered spontaneously [6, 7]. However, severe or long-term ischemia can lead to infarctions at the ischemic site. Although reperfusion can restore blood flow to the ischemic tissues, it often leads to further damage resulting in molecular (lipid, protein, and DNA) damage, cell necrosis, and tissue dysfunction [8–10]. Ischemia-reperfusion (I/R) injuries often occur following postischemia treatments (e.g., relieving coronary artery spasm, dredging the microcirculation in shock, and cardiopulmonary cerebral resuscitation) or surgery (arterial bypass grafting, thrombolytic therapy [11], and organ transplantations [12]). Furthermore, I/R injuries not only harm the I/R site but can also injure other organs and systems. For example, a liver I/R injury not only damages the liver itself but also triggers remote organ dysfunction to the lungs, kidneys, and myocardium [13–15]. Therefore, it is necessary to identify natural active substances that can reduce the impact of ischemic and I/R injuries. Prior studies have shown that several bioactive substances (valsartan [16], carbon monoxide [17], hydrogen sulfide [18], oleuropein [19], and necrostatin-1 [20]) can be used as adjunct therapies for ischemic and I/R injuries. Besides, lycopene has been found to be protective effects on ischemic and I/R injuries.

## 2. Lycopene

### 2.1. Sources and Structure

Lycopene has been isolated and identified for more than one hundred years. As a natural pigment, lycopene is mainly present in red fruits and vegetables, for example, watermelon, papaya, red carrot, and pink guava [21]. Photosynthetic bacteria can also produce a certain amount of lycopene. Lycopene consists of eight isoprene units that contains eleven conjugated double bonds and two nonconjugated double bonds. As a member of the carotenoid family, lycopene exists in the all-*trans* form and can be *cis*-isomerized by thermal energy, light energy, and chemical reactions [22]. 5-*cis*, 9-*cis*, 13-*cis*, and 15-*cis* are the most common *cis* isomers of lycopene. Of these, 5-*cis* lycopene has the highest stability and antioxidation. Although the stability of all-*trans* lycopene is second only to 5-*cis* lycopene, it has the lowest antioxidant properties [23].

**Figure 1:**
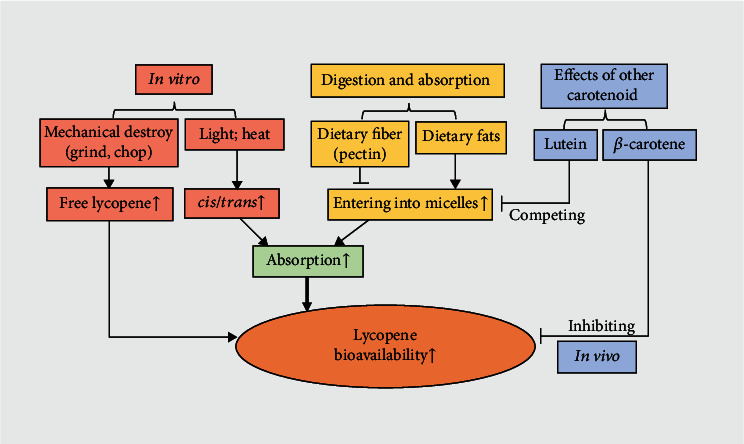
Factors affecting the bioavailability of lycopene. In vitro, mechanical treatments (grinding and chopping) can release more lycopene from food matrix and increase the amount of free lycopene; light-induced and temperature-induced isomerization from all-tran*s* to all-cis can improve the proportion of cis-lycopene isomer. Dietary lipids promote the formation of lipid micelles, allowing more free lycopene into the micelles to aid in its absorption. Dietary fiber (pectin) destroys the formation of micelles, blocking lycopene entering the micelle. In vivo, lutein competes with lycopene into chylomicrons, preventing lycopene into the chylomicrons and reducing the absorption of lycopene; *β*-carotene reduces the bioavailability of lycopene through inhibiting the utilization, catalysis, and metabolism of lycopene.

The bioavailability and biological properties of lycopene are affected by the conversion of *trans*-lycopene to *cis*-lycopene during food processing (e.g., cooking), digestion, and absorption (Figure 1) [24]. For instance, dietary fiber and *β*-carotene impede lycopene absorption, but dietary fats favor it [25]. In micelles, the *cis* isomer of lycopene is superior to the *trans* isomer in solubility, and after entering the intestinal cells through passive diffusion and cholesterol membrane transporters (scavenger receptor class B type I, SR-BI) [26, 27], the *cis*-lycopene isomer preferentially merges into chylomicron particles, enters the lymphatic system, and is released into the bloodstream [28, 29]. Due to the lipophilic nature of lycopene, the concentration of lycopene in adipose tissue is higher than in the serum [30]. In the serum, lycopene is concentrated in low-density lipoproteins (LDL) and very low-density lipoproteins (VLDL) [31]. The testis, adrenal glands, liver, and prostate are also storage sites for lycopene [32, 33]; in these tissues and organs, the concentration of *cis*-lycopene is mildly higher than that of the *trans* isomer.

### 2.2. Biological Functions of Lycopene

#### 2.2.1. Biological Effects of Lycopene

**Figure 2:**
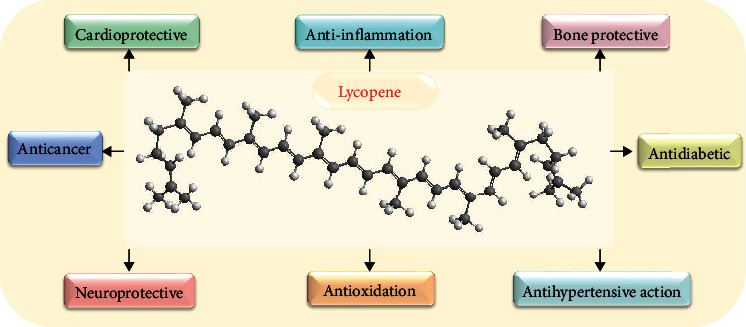
Biological activities of lycopene.

Although lycopene is not an indispensable nutrient for humans, it is still a hot topic in health care. This is mainly because lycopene is ubiquitous in our daily diet and has a significant biological effect on the human body. Multiple kinds of studies (e.g., epidemiological studies, animal studies, human clinical studies, and intervention studies) have substantiated that lycopene has a biological influence on almost every organ and tissue. Moreover, many studies have shown that lycopene administration can prevent and treat many diseases, including cancer [34], cardiovascular disease [35], osteoporosis [36], male infertility [37], obesity and diabetes [38], neurodegenerative diseases [39], rheumatoid arthritis [40], skin [41], and retinal diseases [42] (Figure 2). Because lycopene does not have a b-ion ring structure, it does not have provitamin A activity [43]. Instead, lycopene exerts its biological effects primarily through antioxidation and non-antioxidation. As a highly effective antioxidant, lycopene can decrease the risk of disease by neutralizing reactive oxygen species (ROS) and reducing oxidative damage of some biomolecules (DNA, proteins, and lipids) [44, 45]. In addition, lycopene has also acted through other mechanisms, including anti-inflammation [40], autophagy [46, 47], apoptosis [48, 49], proliferation [50, 51], and pyroptosis [52].

#### 2.2.2. Dietary and Safety

Apart from dietary intake, lycopene had been sold over the counter as a health-care product to prevent diseases. There are different opinions on the recommended dosage and administration time of lycopene for different health purposes. The 67th meeting of the Joint Committee of Experts of the World Health Organization and the Food and Agriculture Organization of the United Nations (FAO) established a permissible daily intake of lycopene of 0–0.5 mg/kg b.w. in healthy people. However, more lycopene may be needed to prevent some diseases. One human study suggested that intake of 6.5 mg lycopene reduced prostate cancer risk in men [53]. Another research reported that increasing the intake of lycopene to 7 mg/d improved the endothelial function of patients with cardiovascular disease [54]. Besides, side effects should be considered when using lycopene to prevent diseases. An animal study testing the safety of two synthetic lycopene products at different concentrations (0, 500, 1500, and 3000 mg/kg b.w./d) found that rats ingesting lycopene at the highest concentration (3000 mg/kg b.w./d) for 13 weeks did not develop any adverse effects [55]. Few side effects have been reported in humans unless a large quantity of tomatoes or lycopene are ingested. Meanwhile, one study reported that after ingesting two liters of tomato juice per day for several consecutive years, one 61-year-old woman developed lycopenemia, but no other adverse events were observed [56]. Three weeks after she stopped ingesting lycopene, the orange color of her skin faded. Although supplementation with a low-dose of lycopene might yield beneficial effects (such as preventing preeclampsia [57]), it should be used carefully by women who are pregnant or lactating. In summary, lycopene is a relatively safe functional ingredient at most doses.

## 3. The Effects of Lycopene on Ischemic and I/R Injuries

### 3.1. The Effects of Lycopene on Myocardial Ischemic and I/R Injuries

The primary cause of global human death is ischemic heart disease which has attracted significant research attention [58]. Ischemic heart disease is brought about by several reasons, including thrombosis, coronary artery stenosis, and excessive contraction of the coronary artery [59]. Myocardial ischemia leads to inadequate oxygen and energy for supporting normal heart function. Besides, long-term or severe cardiac ischemia can result in arrhythmia, myocardial infarction, and sudden death. Mechanical and drug interventions, such as angioplasty, thrombolytic therapy, and coronary artery bypass grafting, can quickly restore blood flow to the ischemic site [60–63]. However, these treatments not only fail to reduce the area of the infarction but can cause more serious damage to the ischemic site (increasing area of infarction and death such as through I/R injury) [64, 65]. Myocardial I/R injuries are extremely complex pathologic processes that are related to various mechanisms, including oxidative stress, inflammation, and apoptosis [66, 67].

The two cardioprotective strategies able to reduce the potential for myocardial I/R injury are preconditioning (IP) and postconditioning (PostC). These strategies can not only lessen the myocardial infarction size and arrhythmia but also improve the myocardial systolic function after ischemia-reperfusion [68–70]. Moreover, some substances, such as lycopene, can be used as adjuvant therapy to mitigate myocardial I/R injury through their antioxidant abilities [71–75]. However, if antioxidants are used indiscriminately to quench reactive species (ROS/RNS) during I/R injury, they may interfere with normal physiological redox signals and damage cellular function [76]. For example, prior studies showed that during the trigger stage of IP and PostC, the production of ROS/RNS was beneficial to activating protein kinase C (PKC), forming memory effects, and inducing the RISK (reperfusion injury salvage kinase) and SAFE (survivor activating factor enhancement) pathways to inhibit the opening of mPTP during reperfusion [77–79]. Antioxidants given during this phase would block the cardioprotective effects of IP and PostC [80, 81]. Therefore, the appropriate time for supplying antioxidants should be determined in consideration of the active phase of ROS/RNS by taking into full consideration its dual role in myocardial I/R injury. Moreover, myocardial I/R injuries can be aggravated (through increasing ROS/RNS and reducing antioxidant enzymes) by the presence of comorbidities (e.g., myocardial hypertrophy, diabetes, and metabolic syndrome [82–86]). Increased doses of antioxidants should be considered to achieve a better therapeutic effect in these patients. Thus, when lycopene is given as an adjunctive therapeutic measure, it is essential to identify the best dose and time of administration for this drug.

#### 3.1.1. Lycopene Attenuates Myocardial Ischemic Injury

Prior epidemiological studies reported that lycopene might exert a protective effect against myocardial infarctions (MI). According to a cross-sectional study [87], the level of lycopene in serum was negatively related to the risk of an acute MI in men. Meanwhile, according to the case-control study, it could be found that in the adipose tissues, higher levels of lycopene were significantly correlated with a lower risk of MI [88]. Because lycopene concentration in adipose tissues can better reflect its long-term accumulation than serum concentration, this result more reliably and accurately reflects the correlation between high levels of lycopene and a low risk of MI. Further, it also hints that maintaining high levels of lycopene *in vivo* may prevent myocardial ischemia. In addition, lycopene may also lower the risk factors for myocardial ischemia. An epidemiological study found that in the early stages of atherosclerosis, serum lycopene concentration was negatively associated with common carotid intima artery media thickness [89]. Based on these studies, it can be found that high levels of lycopene in the body appear to not only directly prevent MI but also reduce the impact of one of its primary risk factors (atherosclerosis). However, it is worth doing more clinical studies to explore the specific effects and related mechanisms of lycopene in preventing MI.

Some recent studies using animal ischemic models showed that supplying lycopene before and after ischemia could improve the consequences of an ischemic MI (Table 1). It is well known that adrenaline and isoprenaline (ISP) are usually used to induce animal myocardial ischemia. Adrenaline and ISP produce free radicals, inducing MI through autooxidation and positive inotropic and chronotropic effects. Moreover, this MI model better simulates human MI pathophysiology, biochemistry, morphology, functional changes, and histopathology [90–92]. The cardioprotective effects of lycopene in this MI model were related to anti-inflammation (reducing IL-1*β*, IL-6, TNF-*α*, and NF-*κ*B); antioxidation via increasing antioxidant enzymes activities (SOD, GPx, CAT, and GSH) and decreasing the level of ROS; antiapoptosis by increasing Bcl-2 and reducing Bax, Cyt-c, caspase-3, caspase-8, caspase-9, the ratio of Bax/Bcl-2, and JNK/(ERK1, STAT1,3); promoting repair (inhibiting eight miRNAs and increasing IGF-1 and SIRT-1); and reducing remodeling (inhibiting p38/MMP-9) [93–99]. Besides, other studies have shown that quercetin can be used in combination with other antioxidants to protect against ischemic injury [100, 101]. The combined application of lycopene and quercetin improved myocardial ischemic injuries in a rat model [102]. In contrast with quercetin treatment (100 mg/kg b.w.) alone [103], lower doses of quercetin administration (80 mg/kg) combined with low-dose lycopene (3 mg/kg) could achieve similar protective effects (restoring the ischemic injury of myocardial to normal status). This combination could also shorten administration time (10 d) compared with quercetin alone (30 d). Therefore, in-depth exploration of additional synergies between lycopene and other agents should be pursued in order to achieve the best therapeutic effects.

#### 3.1.2. Lycopene Attenuates Myocardial I/R Injury

Many studies have discussed lycopene in protective effects on myocardial I/R injury. The only published randomized controlled clinical study reported that after standard MI treatment (giving aspirin, statin, and *β*-blocker), lycopene administration (30 mg 12 hours before PCI and 15 mg before and 8 hours after the procedure) significantly reduced the serum CK-MB levels of 28 patients [104]. The findings showed that lycopene had the potential to prevent cardiovascular events after a myocardial I/R injury. However, lycopene supplementation had no significant effect against increases in serum TnI and hs-CRP levels. This was counter to the findings of other studies, which showed that lycopene could lower CRP levels in both heart failure patients and healthy individuals [105, 106]. These contrasting results may be because the randomized trial used lower dose of lycopene for a shorter period.

Many studies (*in vivo* and *in vitro*) using animal I/R models showed that lycopene supplementation before and after ischemia could significantly improve myocardial I/R injuries (Table 2). Relevant mechanisms include antioxidation (inhibiting ROS production; increasing GSH, SOD, CAT, and GSH-Px activities; and activating AMPK-ACC pathway), enhanced autophagy (improving p-AMPK, MAP1LC3B, and Beclin 1), anti-inflammatory effects (reducing IL-6, TNF-*α*, and IL-1*β* and weakening JNK pathway), ER stress inhibition (reducing the mRNA expression of eIF2*α*, GRP78, CHOP, ATF6, caspase-12, and sXbp-1 and the phosphorylation of AMPK and eIF2*α*), and reduced mitochondrial apoptosis (increasing Tfam, Bcl-2, and the phosphorylation of Akt and ERK1/2; inhibiting the opening of mPTP; and decreasing the level of Cyt-c, Bax, APAF-1, cleaved caspase-3 and caspase-9 and p-GSK-3*β*, and the ratio of Bax/Bcl-2,). Besides, it could be found that these protective effects were produced by long-term low-dose administration or a single high-dose lycopene. Although lycopene dosage in this study [107] was too high to unsuitable for clinical applications compared with the international intake standard of lycopene for healthy people (0–0.5 mg/kg/d b.w.) [53, 54], the study did not find any toxicity from the use of higher concentrations of lycopene. However, whether such concentrations could be used clinically requires further investigation. Further, some studies showed that high cholesterol not only exacerbated myocardial damage but also affected the therapeutic effects of PostC [108–112]. The combination of PostC and a certain dose of lycopene could significantly improve severe myocardial I/R injuries in patients with high cholesterol [113], justifying further exploration of potential combination therapies in myocardial I/R injuries under other conditions (e.g., diabetes and hyperlipidemia [114, 115]).

Furthermore, one study found that the cardioprotective effects of IP and PostC were activated through the posttranslational modification of proteins (S-nitrosylation, SNO) mediated by RISK and SAFE [116]. Most of the evidence suggests that as an adjunctive therapeutic measure, lycopene acts on many downstream signal molecules of RISK and SAFE (e.g., activating ERK, Akt and PI3K and inhibiting STAT3 and p-GSK-3*β*) to attenuate myocardial I/R injury [113]. These studies implied that lycopene seemed to promote SNO. Moreover, the activity of antioxidant enzymes (GSH and SOD) could be improved by lycopene's cardioprotective effects [97]. These antioxidant enzymes could also limit protein SNO levels through denitrosylation, thereby preventing excessive SNO from inducing cytotoxicity in the pathological environment created by nitrosative stress [117–119]. Therefore, lycopene may promote SNO and inhibit the production of excess SNO to alleviate myocardial I/R injury. Nevertheless, further studies are still required on this topic.

### 3.2. Lycopene Attenuates Liver I/R Injury

In a variety of clinical settings (e.g., hemorrhagic shock, hepatic resection, liver transplantation, and trauma [128–130]), liver I/R injury is prone to occur. Liver I/R injury not only damages the liver itself but also triggers the remote organ dysfunction to the lungs [13], kidneys [14], myocardium [15], and intestines [131]. This effect has made liver I/R injury prevention a hot research topic in recent years. As the liver is the main storage site of lycopene, it is highly likely that lycopene acts on the liver. Many studies have shown that lycopene could effectively impact liver-related diseases, including cirrhosis [132], nonalcoholic fatty liver [133], and hepatocellular carcinoma [134].

A recent epidemiological study compared changes in the plasma antioxidant levels (vitamin A, vitamin E, *β*-carotene, and lycopene) of the 12 patients who underwent liver transplantation (liver I/R) at three stages of their transplant (preanhepatic, anhepatic, and reperfusion) [135]. The levels of lycopene and *β*-carotene declined much faster than those of vitamin A and vitamin E. This decrease may be related to the reduction or disappearance of the liver's ability to store lycopene in the setting of liver disease. Lipid peroxidation also increased during reperfusion, possibly due to the almost complete depletion of lycopene in the plasma, and hints that lycopene may be essential to maintaining oxidative balance during liver transplantation. It is therefore possible that lycopene supplementation during hepatic I/R injury (e.g., liver transplantation) could significantly reduce oxidative damage during I/R.

Prior animal studies showed that lycopene pretreatment could effectively mitigate hepatic I/R injury by enhancing the Kupffer cell autophagy (activating Nrf-2/HO-1) and inhibiting inflammation (decreasing IL-6, TNF-*α*, IL-1*β*, and NLRP3 inflammasomes and increasing IL-10), oxidative stress (reducing ROS and increasing CAT), and apoptosis (increasing Bcl-2 and decreasing caspase-1 and caspase-3) (Table 3). One study showed that apart from CAT, ALT, AST, LDH, and MDA levels were reduced by lycopene pretreatment in a dose-dependent manner [136]. According to these results, the protective effects of lycopene on hepatic I/R injury might be associated with the antioxidant capacity of lycopene or the activation of the Nrf2 pathway [137]. Some studies are worth exploring whether lycopene pretreatments can improve hepatic I/R injury-induced damage to other organs.

### 3.3. Lycopene Attenuates Kidney I/R Injury

Some urological procedures (e.g., kidney transplantation, partial nephrectomy, and renal artery surgery) [140] and crisis situations (trauma, hemorrhagic shock, and sepsis) [141] can induce renal ischemia and acute kidney injury. Subsequent reperfusion induces neutrophil activation and the release of ROS and other inflammatory mediators [142, 143], leading to decreased tubular and glomerular function and further exacerbates kidney damage [144]. Exploring effective measures to reduce renal I/R injury is critical.

Encouragingly, some studies have shown that lycopene pretreatment could effectively improve the histopathologic and biochemical changes induced by renal I/R injury via antioxidation and anti-inflammation. A recent study found that before renal I/R injury (30 min/2 h), intake of lycopene (at 30 min before ischemia, 10 mg/kg, i.p.) reduced the levels of urea and creatinine in the serum and improved pathologic changes to renal tissues and cells by inhibiting the Notch2/Hes-1 pathway; decreasing Bax, IL-6, and TLR-2; and increasing Bcl-2 [145]. Another study showed that in the renal I/R injury (45 min/6 h), lycopene pretreatment (at 30 min before ischemia, 100 mg/kg, i.g.) reduced serum MDA levels and histopathologic changes [146]. However, serum BUN and creatinine levels, tissue MDA level, and the GSH levels in the serum and tissues did not significantly change. An additional study showed that the before kidney I/R injury (45 min/24 h) [147], lycopene supplementation (4 mg/kg/d, for 2 d before ischemia) significantly improved sodium, GSH, and CAT levels in addition to pathology scores. However, it did not change the levels of other indices (potassium, creatinine, BUN, MDA, SOD, and GSH-Px). Based on these studies, the duration of ischemia appears to affect lycopene in renal I/R injury protective effects beyond dose and pretreatment length. In view of lower-dose lycopene pretreatment in renal I/R injuries protective effects only following short-term ischemia, higher-dose regimens before and after renal I/R injury following long-term ischemia should be evaluated.

### 3.4. Lycopene Attenuates Cerebral Ischemic and I/R Injuries

Cerebral ischemia occurs in the setting of brain damage, asphyxia, and cardiac arrest [148]. During cerebral ischemia, the release of cytokines, mitochondrial respiratory enzymes damage, programmed cell death induction, and microglia activation can lead to irreversible brain damage [149–151]. Although timely reperfusion can mitigate these injuries by arresting these pathologic changes, activated inflammation and oxidative stress after reperfusion destroy the blood-brain barrier and induce the death of neuronal [152]. Because lycopene can cross the blood-brain barrier and directly act on neurons [32], lycopene has been used to improve cerebral disease effects (subarachnoid hemorrhage [153], Alzheimer's disease [154], memory impairment [155], and central and peripheral neuropathy [156]). Recent studies have shown that lycopene can not only improve cerebral ischemia but also reduce cerebral I/R injury.

Only one epidemiological investigation and one *in vitro* study have explored the protective effects of lycopene against cerebral ischemic injuries. In a prospective follow-up study, Karppi et al. [157] found that the high serum concentrations of lycopene were closely associated with a reduction in the risk of ischemic stroke in men. It suggested that lycopene might play a vital role in preventing ischemic stroke. *In vitro*, the trial found that lycopene pretreatment (0.5, 2.0, and 8.0 *μ*mol/L, 1 h) could prevent oxygen-glucose deprivation- (OGD-, 3, 6, 12, and 24 h) induced SH-SY5Y cell death by reducing autophagy mediated by activated AMPK/mTOR following the inhibition of oxidative stress (increasing xCT, cysteine, and GSH levels and decreasing the levels of ROS and MDA) [158]. It is worth conducting additional clinical investigations and animal studies to explore the specific protective effects and mechanisms of lycopene against cerebral ischemic and I/R injuries.

Several animal studies showed that lycopene supplementation before and after ischemia and after reperfusion could reduce neuronal apoptosis and cerebral infarction area (Table 4). Lycopene's mechanism of action is related to anti-inflammation (inhibiting IL-6/JAK-STAT3), antioxidation (increasing SOD, GSH, and CAT activities and the mRNA expression of HIF-1*α*; reducing ROS, iNOS, and NO; and activating Nrf-2/HO-1), antiapoptosis (reducing caspase-3, JNK/MAPK, and Bax and increasing Bcl-2), and lowering iron content (raising FNP1 and decreasing hepcidin and L-ferritin). Nanoliposome-encapsulated lycopene (L-LYC) was more effective at preventing a brain I/R injury than naked lycopene [159]. This might be because L-LYC more easily crosses the blood-brain barrier, thereby increasing lycopene content in the brain. Among currently known nanoparticles, gold nanoparticles (GNPs) have been of particular interest because of their unique surface chemical properties, excellent biocompatibility, and potential for extensive chemical modification [160, 161]. Some studies found that GNPs played an essential role in treatments (20 nm GNPs remarkably exhibited neuroprotective effects, while 5 nm GNPs aggravated neuronal damage) [162] and diagnosis (GNPs recognized the infarct area of an ischemic stroke by combining with various biological molecules such as hyaluronic acid) [163]. Further, modifying GNPs with some biomolecules could transport nanoparticles to specific locations [164]. To permit the accurate transport of the appropriate dose of lycopene to sites of infarction and improve its therapeutic effects, it may be possible to modify GNPs with some biomolecules (directionally transported to the brain) as carriers instead of using naked GNPs. However, it is important to consider whether modifying GNPs with biomolecules will affect their diagnostic and treatment potential.

### 3.5. Lycopene Attenuates Reproductive Organ I/R Injuries

The ischemia of the reproductive organs largely occurs in the testicles of males and the ovaries of females. Testicular torsion is the primary cause of testicular ischemia and commonly occurs in neonates and adolescents [169, 170]. Ovarian ischemia is usually caused by ovarian torsion, surgery, and pregnancy [171]. Long-term ischemia of the testis and ovaries can lead to cell damage and declined function, resulting in hormone secretion disorders, low fertility, and infertility [172]. Severe pain due to torsional ischemia requires a timely operation to detorsion of the organ, relieve the patient's pain, and restore blood flow. Although reperfusion is highly necessary to the survival of ischemic tissues, reperfusion also aggravates damage to these reproductive organs. It is therefore equally important to restore blood supply to reverse the ischemic injury and to take measures to reduce reperfusion injury.

Fortunately, current studies had shown that lycopene treatment could protect the reproductive organs from I/R injury. Following testicular I/R injury (1 h/3 h, 24 h, 30 d), lycopene intake (4 mg/kg/d, i.g.) after 5 min of ischemia effectively improved bilateral testicular sperm motility and decrease oxidative stress [173]. However, recent research [174] showed that lycopene supplementation (20 mg/kg/d, i.p.) at 2 h after the ischemia could only reduce germ cell apoptosis following a short-term testicular I/R injury (2 h/3 d) and did not protect against long-term I/R injury (2 h/10 d). According to these studies, it can be found that for short-term ischemia, the supplement with lycopene significantly alleviates testicular I/R injury. However, even increasing lycopene concentration failed to improve the damage caused by long-term ischemia. Furthermore, the longer the testicular reperfusion time, the worse the therapeutic effect of lycopene. This suggests that timely administration of high-dose lycopene can be used as an adjunctive therapy against testicular I/R injury. Moreover, it also suggests that an evaluation about the benefits of lycopene pretreatment against testicular I/R injury is warranted. Further epidemiological investigations should focus on whether patients have milder symptoms of I/R injury after taking more lycopene.

Besides, lycopene has also been shown to improve ovarian I/R injury. One study [175] showed that at 2.5 h after ischemia (3 h), lycopene treatment (100 and 200 mg/kg, i.g.) could effectively reduce tissue congestion, hemorrhage, and polymorphonuclear leukocyte infiltration via increasing GSH and CAT and lowering MDA and MPO. Moreover, the protective effects of lycopene were dose-dependent. Another study showed that lower doses of lycopene (before 1 h of reperfusion, 2.5 and 5 mg/kg, i.p.) could reduce ovarian tissue degeneration, vascular congestion, inflammatory cell infiltration, and edema [176]. However, there was no difference in other pathological indexes (MDA and p-NF-*κ*B) between the two different doses. The differing results of these dose-effect studies are likely due to different dose designs. On one hand, the dose of the former was higher than that of the latter. On the other hand, the dose difference in the former study was also relatively larger, which may result in significantly different effects. Further studies are therefore needed to identify the lowest doses of lycopene required to achieve the best therapeutic effects in different I/R circumstances.

### 3.6. Lycopene Attenuates the Intestinal Tract I/R Injury

Intestinal ischemia is often seen during the progression of some diseases, for example, volvulus [177], acute mesenteric ischemia [178], neonatal necrotizing enterocolitis [179], trauma [180], and intestinal transplant rejection [181]. As a pathologic process, intestinal ischemia can cause intestinal mucosal barrier and bacterial translocation (BT) [182]. With prolonged ischemic time, intestinal ischemia can lead to irreversible intestinal tissue necrosis and serious systemic metabolic derangement [182]. Although reperfusion can repair some mild injuries and prevent irreversible damage [9], it can aggravate intestinal injuries, leading to shock, multiple organ dysfunction, and sepsis. Although new therapies have been proposed, the morbidity and mortality from intestinal I/R injuries remain high [183]. The study about lycopene in effects on intestinal I/R injury had just begun. A prior research showed that lycopene pretreatment (12 h before ischemia, 0.4 mg/kg, i.g.) could significantly protect the intestinal villi structure from necrosis, degeneration, and spillage of the epithelium, thereby reducing IgA and sIgA loss and limiting BT [184]. These effects were related to anti-inflammation and the maintenance of the tight junction via activation of retinoic acid receptor- (RAR-) mediated transcriptional pathways. However, lycopene intake (0.4 mg/kg, i.g.) 1 h after reperfusion could not protect the intestine from I/R injury (30 min/12 h). Those results suggest that low-dose preconditioning using lycopene could effectively prevent intestinal I/R injury, but treatment after reperfusion might require higher doses to achieve a therapeutic effect. Moreover, the intestinal IgA, as everyone knows, plays a crucial role in mucosal immunity and infection by binding with pathogens to arrest them from crossing the epithelial barrier [185, 186]. According to the result that lycopene could maintain the intestinal IgA level as shown in that prior study, it may be useful for alleviating gastrointestinal symptoms (anorexia, nausea and vomiting, and diarrhea) and impaired nutrient absorption [187, 188] in patients suffering from intestinal mucosal and gut microbiome dysfunction following COVID-19 infection. However, in order to confirm this hypothesis, more research is needed.

### 3.7. Lycopene Attenuates Retinal I/R Injury

Retinal ischemia occurs during many pathologic processes, including glaucoma, diabetic retinopathy, and retinal vascular obstruction [189, 190]. As a high oxygen-consuming organ, the retina is extremely sensitive to ischemia [191]. Mild ischemia increases the retina's vascular permeability, leading to edema. With increasing severity, the retinal injury gets worse, causing retinal ganglion cell apoptosis, reduced vision, and blindness [192, 193]. Timely recovery of blood flow is particularly vital to reverse a retinal ischemic injury. However, reperfusion can cause a series of pathologic problems (e.g., oxidative stress, inflammation, and apoptosis) that can exacerbate the damage of ischemic retinal [194–196]. Studies have shown that lycopene may have potential applications in prophylaxis and therapy against cataract and diabetic optic neuropathy through its antioxidative and anti-inflammatory effects [197, 198]. A recent study explored the effects and mechanisms of lycopene on retinal I/R injuries i*n vivo* and *in vitro* [42]. *In vivo*, it was found that lycopene treatment (40 *μ*M, injected into the ocular bulbs) before retinal I/R injury (3 h/24 h) could prevent cell morphologic changes and reduce apoptosis by activating the KEAP1/Nrf2/ARE pathway. Besides, *in vitro*, different doses of lycopene (10 *μ*M and 20 *μ*M, for 24 h) culturing cells before OGD/reperfusion (40 min/2 h, 4 h) could reduce ROS and MDA and increase cell viability by enhancing antioxidant enzyme activity (GSH, CAT, and SOD) and activating the KEAP1/Nrf2/ARE/(HO-1, NQO-1) pathways. While low-dose lycopene treatment had no obvious protective effects against long-term retinal I/R injury, high-dose lycopene had significant protective effects against both short- and long-term I/R injuries. Higher doses of lycopene are therefore required to reverse retinal I/R injuries.

### 3.8. Lycopene Attenuates Spinal Cord I/R Injury

Spinal I/R injury occurs mostly after thoracic aorta, common thoracic, and abdominal artery surgeries [199]. One of the most serious complications of these surgeries is spinal I/R injury, which has a very poor prognosis [200]. Spinal I/R injury can seriously impair nerve function and lead to paraplegia [200]. One survey showed that the incidence of a spinal I/R injury during thoracic aortic surgeries worldwide was approximately 1 in 10 [201]. However, the therapeutic effects of currently known interventions (e.g., cerebrospinal fluid drainage [202], electro-needle pretreatment [203], drug administration [204], multifunctional hepatocyte use [205], remote ischemia [206], and hyperbaric oxygen pretreatment [207]) are unsatisfactory. Therefore, it is urgent to find better interventions for preventing spinal I/R injury. A recent study showed that after a spinal I/R injury (40 min/48 h) [208], lycopene (25 and 50 mg/kg/d, for 14 d, i.g.) could significantly improve neuroprotection and reduce motor dysfunction, apoptosis (increasing the neuron survival rate), and inflammation (decreasing IL-8, TNF-*α*, IL-1*β*, and IL-6). These effects were related to the downregulation of COX-2 expression via HO-1 activation. Besides, the protective effects of the high-dose lycopene were significantly better than those of low-dose lycopene. Although the study showed that lycopene could lessen spinal I/R injury, lycopene pretreatment was preferred. Further studies should therefore focus on the lycopene pretreatment in protective effects against spinal I/R injuries.

### 3.9. Lycopene Attenuates Limb I/R Injury

Limb muscle ischemia often occurs in some clinical settings, including thrombosis [209], limb surgical revascularization, trauma [210], and free flap reconstruction [211, 212]. During early ischemia, the limb gets cold, numb, and painful. As the ischemia time continues, the limb's skin color changes to cyan/black and skeletal muscle necrosis occurs. Further worsening of ischemic injuries can lead to amputation and death. Common reperfusion treatments include intravascular interventional surgery [213], bypass surgery [214], hybrid reconstruction [215], and cell therapy [216]. However, reperfusion leads to metabolic waste that accumulated in the ischemic limb reentering the systemic circulation, which may result in additional organ injury, multiple organ failure, and death [217]. One previous study showed that after strenuous exercise, lycopene could alleviate oxidative stress to protect the skeletal muscle by reducing xanthine oxidase and myeloperoxidase activity [218]. It is therefore possible that lycopene could reduce skeletal muscle I/R injury. A previous study showed that lycopene (10 mg/kg/d, for 15 d, p.o.) intake prior to rat limb muscle I/R injury (2 h/2 h) [219] significantly reduced muscular atrophy, perivascular inflammation, and focal central muscle necrosis by decreasing MDA and IMA and increasing GSH-Px and SOD activities. In the serum and muscle tissue, MDA levels both decreased significantly, and the average level of MDA in the muscle was lower than that in the serum. This suggests that the antioxidant effects of lycopene on skeletal muscle were better than in the serum. However, it did not impact lycopene's positive impact on other organs (even distant organs [220, 221]) injured following a limb I/R injury. In a recent study, the results showed that lycopene pretreatment (10 mg/kg/d, for 15 d, p.o.) could prevent lung injury (induced by skeletal muscle I/R injury, 2 h/2 h) via increasing SOD and GPX and reducing MDA [222]. It can be hypothesized that lycopene may prevent single organ I/R injuries from affecting other organs. However, it is unclear whether the protective effects and mechanisms of lycopene are the same in different organs. Before clinical application, further research is necessary.

### 3.10. Lycopene Attenuates Ischemic Injury to Mesenchymal Stem Cells

As a kind of multifunctional stem cells, mesenchymal stem cells (MSC) can differentiate into various types of terminal cells, including stem cells, cardiomyocytes, and neurons. So MSC transplantation has been used to treat tissue damage such as limb ischemia [223], acute kidney injury [224], liver fibrosis [225], ischemic heart failure [226], myocardial infarction [227], and the I/R injuries to the heart [228], kidney [229], liver [230], spine [231], and brain [232]. However, the therapeutic effects of MSCs are limited by their low implantation rate, low survival rate, impaired paracrine ability, and delayed administration [233]. Moreover, previous studies showed that the low survival rate of MSCs was due to oxidative stress, apoptosis, and autophagy caused by ischemia [234–236]. Therefore, it seems feasible to improve the implanted cells survival rate through protecting MSC from oxidative stress, apoptosis, and autophagy. A study showed that before hypoxia (6 h), precultured cells with lycopene (20 *μ*M, for 1 h) could activate the PI3K/Akt pathway to inhibit the production of ROS and reduce apoptosis [237]. Additionally, in a different ischemia model (low glucose and H_2_O_2_ treatment, 1, 2, 3, and 4 h), lycopene pretreatment (10 *μ*g/mL, for 30 min) significantly inhibited oxidative stress (reducing ROS levels and activating Akt-MnSOD) and apoptosis (decreasing the levels of p-p38, p-ATM/p53, Bax, and p-JNK and increasing Bcl-2) [238]. According to these studies, it can be found that lycopene may protect MSCs from ischemic injuries. However, there are currently no studies that evaluate lycopene in the protective effects on MSC I/R injury. Therefore, it is essential to further explore the effects and mechanisms of lycopene in the prevention and treatment of MSC I/R injury, in order to improve the MSCs survival rate during transplantation.

### 3.11. Lycopene Attenuates Bladder I/R Injury

The main functions of the bladder are to store urine under low pressure and discharge urine via coordination with nerves and muscles. However, some conditions (e.g., bladder outlet obstruction, ligation, complications from pregnancy or pelvic surgery, compression by pelvic tumors, and lower urinary tract obstruction [239–241]) can lead to bladder hypoperfusion, resulting in ischemia. Due to ischemia, the structure and function of the bladder will be altered, which can cause noncompliance and hyperreflexia [242, 243]. Therefore, blood flow must be restored as soon as possible. However, the damages occurring in these areas (nerves, synapses, and smooth muscle cells) are further aggravated when oxygen-rich blood quickly enters the ischemic bladder [244, 245]. Reperfusion can lead to neurologic injury, hypercapnia, and irregular and decreased contractile responses [246–248]. Although no studies have directly shown that lycopene could improve bladder I/R injury, a new type of antioxidant complex containing the lycopene chemical structure has been found to reduce detrusor nerve injury in a guinea pig whole urinary bladder *in vitro* during bladder H/R injury (1 h/2 h) better than a substance containing the chemical structure of vitamin C [249]. Through these results, it can be found that lycopene exerting the neuroprotective effects in the bladder may not relate antioxidant activity, as vitamin C has better antioxidant properties than lycopene. This finding warrants further investigation.

## 4. Mechanisms

### 4.1. The Mechanisms behind Lycopene Attenuation of Ischemic Injuries

**Figure 3:**
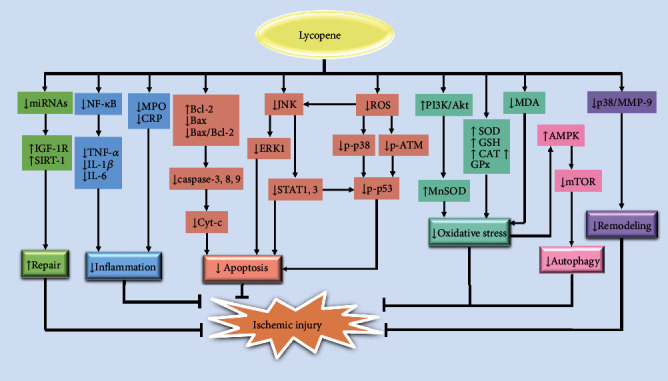
The mechanisms of lycopene attenuating ischemic injury.

Ischemia damages organ function by depriving them of nutrition and oxygen. Long-term or severe ischemia can lead to acute pain, infarction, and sudden death. Once the infarct is formed, the currently known interventions such as thrombolytic therapy, angioplasty, and percutaneous coronary intervention can restore blood flow and relieve ischemic pain but have a lesser ability to reduce the infarct area. Adjuvant treatments that can potentially improve the efficacy of the above interventions should therefore be considered. Many studies have shown that lycopene supplementation before and after ischemia can significantly reduce ischemic injuries (e.g., infarct area, cell necrosis, edema, and inflammatory infiltrates) to some organs. Most of these studies focused on myocardial ischemia, while fewer focused on MSC and brain ischemia. The effects of lycopene are made by inhibiting oxidative stress, apoptosis, and inflammation. Moreover, lycopene can reduce remodeling in ischemic pathologies and promote tissue repair (Figure 3).

Firstly, lycopene decreases ROS and lipid peroxidation (MDA) levels primarily due to its strong antioxidant properties. In addition, lycopene can also inhibit oxidative stress via increasing the activity of SOD, GSH, GPx, and CAT. However, the Nrf-2 pathway does not seem to participate in lycopene's inhibition of oxidative stress, and the reduced level of Nrf-2 may be the result of restored redox status by lycopene. At the same time, lycopene also activates the PI3K/Akt pathway and further increases MnSOD levels to inhibit oxidative stress. Lycopene's inhibition of autophagy can also reduce ischemic injuries via decreased oxidative stress by activating AMPK and inhibiting mTOR. In addition, lycopene also reduces ischemic injuries by decreasing apoptosis via inhibition of ROS-dependent and non-ROS-dependent pathways. The former includes decreased (p-p38 or p-ATM)/p-p53 and JNK/(ERK1, or STAT1,3) pathways that are induced by ROS. The latter includes direct reductions in the levels of caspase-3, caspase-8, caspase-9, and Cyt-c via increased Bcl-2 levels and decreased Bax levels. Besides, lycopene can also directly inhibit the JNK/(ERK1, STAT1,3) pathways. Moreover, lycopene's anti-inflammatory properties are the result of inhibiting NF-*κ*B/inflammatory factors (TNF-*α*, IL-1*β*, and IL-6), MPO, and CRP. Additionally, reduced levels of miRNAs (29c-3p, 194-5p, 503-5p, 20a-5p, 30a-5p, 192-5p, 30e-5p, and 126-3p) or increased IGF-1 and SIRT-1 levels also affect lycopene's reparative properties. Furthermore, inhibition of the p38/MMP-9 pathway by lycopene also impedes remodeling after infarcts.

### 4.2. The Mechanisms behind Lycopene Attenuation of I/R Injuries

**Figure 4:**
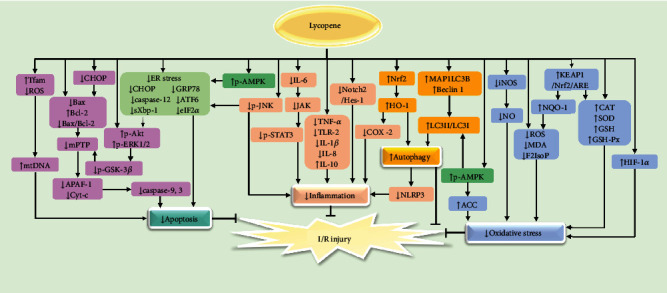
The mechanisms of lycopene attenuating I/R injury.

Timely reperfusion is still essential to mitigating ischemic damage to tissues and organs. However, reperfusion can further aggravate pathologic insults (molecules, cells, and organ dysfunctions). Furthermore, compared with ischemic injuries, I/R injuries are more serious due to their longer-lasting effects and incomplete reversal following reperfusion. Therefore, effective adjunctive therapies are urgently needed. So far, many studies have shown that lycopene administration before and after reperfusion and before ischemia could improve I/R injury to almost all organs (Figure 4). Meanwhile, several mechanisms behind this have been identified, including inhibiting inflammation, oxidative stress, apoptosis, and promoting autophagy.

At first, lycopene inhibits oxidative stress during I/R injuries by directly enhancing the activity of SOD, CAT, GSH, and GSH-Px and reducing the levels of ROS, MDA, and F2IsoP. Lycopene can also indirectly increase NQO-1; decrease ROS, MDA, and F2IsoP levels; and improve the activity of antioxidant enzymes by activating KEAP1/Nrf2/ARE. Upregulated expression of HIF-1*α* mRNA, AMPK/ACC pathway activation and iNOS/NO pathway inhibition by lycopene can also account for its effects on oxidative stress. Secondly, lycopene can also improve the effects of I/R injuries by reducing apoptosis. On one hand, lycopene restores normal mitochondrial function via lowering ROS and raising Tfam. On the other hand, lycopene also directly inhibits caspase-mediated apoptosis and ER stress-induced apoptosis. The former is inhibited not only by lycopene directly but also through reduced ER stress indirectly. Lycopene reduces apoptosis by decreasing p-GSK-3*β* and the ratio of Bax/Bcl-2 (increased Bcl-2 and decreased Bax), thereby increasing p-Akt and p-ERK1/2 while inhibiting the opening of mPTP and preventing Cyt-c from being released into the cytoplasm for APAF-1 binding. The latter is inhibited through reduced ER stress by AMPK activation and JNK inactivation.

Besides, lycopene suppression of JNK can lead to reduced inflammation and inflammatory signaling factors (decreasing TNF-*α*, TLR-2, IL-1*β*, and IL-8 and increasing IL-10). Lycopene can also mitigate inflammation by directly inhibiting the Notch2/Hes-1 and IL-6/JAK-STAT3 pathways. Furthermore, HO-1 activated by Nrf-2 can weaken inflammation not only by inhibiting COX-2 but by increasing Kupffer cell (KC) autophagy and reducing the production of NLRP3. Additionally, lycopene can also promote autophagy by increasing the levels of p-AMPK, MAP1LC3B, and Beclin 1. Although the antioxidant effects of Nrf2 are not explored in this study [137], it can be hypothesized that Nrf2 is most likely involved in oxidative stress inhibition in the setting of I/R after lycopene administration.

## 5. Summary and Prospect

In conclusion, lycopene significantly reduces the impact of ischemic (e.g., infarction) and I/R injuries to multiple organs by inhibiting inflammation, oxidative stress, and apoptosis and promoting autophagy. Lycopene also improves repair and remodeling after ischemic injuries. Besides, lycopene may prevent damage to other organs caused by an ischemic or I/R injury to a different organ. Considering the different nature of ischemic and I/R injuries, a certain dose of lycopene is needed to assure its protective effects against both. For instance, in some special cases (such as high cholesterol [113]), higher doses of lycopene are required in order to achieve better effects. Furthermore, based on previous epidemiological studies, regular monitoring to maintain a certain concentration of lycopene *in vivo* may be a feasible strategy to achieve satisfied effects on preventing ischemic and I/R injuries.

While existing evidence is encouraging, additional studies are required before lycopene can be used clinically. Further clinical studies should focus on lycopene administration method, dosage, and time to achieve optimal protective effects. Additional study should also evaluate if lycopene can improve the damage to other multiple organs caused by ischemic or I/R injuries to one organ and the possibility of using lycopene to simultaneously attenuate ischemic and I/R injuries to multiple organs. Moreover, new drug delivery techniques should be continuously explored to ensure that a certain dose of lycopene can accurately reach the ischemic site, thereby guaranteeing its protective effects.

## Figures and Tables

**Table 1:** Effects of lycopene on myocardial ischemic infarction.

Species	Ischemic models	Treatments	Effects and mechanisms	Ref
BALA/c mice	Ligating LAD	Lycopene (10 mg/kg/d, i.g.) for 4 weeks after ischemia	Relieving myocardial apoptosis and inflammation via reducing the expression of TGF-*β*1, IL-1*β*, NF-*κ*B TNF-*α*, collagen I, collagen III, caspase-3, caspase-8, and caspase-9	[93]
Wistar rats	Coronary artery ligation	Lycopene (1 mg/kg/d) for 3 months after ischemia	Attenuating cardiac remodeling and improving diastolic dysfunction via ↓Nrf-2, TNF-*α* and eight miRNAs, ↑IGF-1R, and SIRT-1	[94]
Wistar rats	At intervals of 24 h, ISP (200 mg/kg, s.c.) for 2 d	Lycopene (10 mg/kg/d, p.o.) for 30 d after ischemia	Reducing infarction area and preventing DNA damage and oxidation of enzyme SH groups via scavenging ROS, ↓caspase-3, iNOS, NO, CRP, and MPO	[95]
Sprague-Dawley rats	Ligating LAD	Lycopene (40 mg/kg/d, i.g.) for 28 d after ischemia	Attenuating ventricular remodeling and LV dilation, improving LV systolic, and cardiac function via inhibiting p38/MMM-9 and the type I collagen	[96]
Wistar rats	ISP (85 mg/kg, s.c.) at 28 and 29 d	Lycopene (0.5, 1.0, and 1.5 mg/kg, i.g.) for 30 d	Improving hemodynamics and disorder of the cell morphology and reducing cardiac dysfunction and via ↑SOD, CAT, GSH-Px and GSH, ↓MDA, CK-MB, and LDH	[97]
Sprague-Dawley rats	ISP (85 mg/kg, s.c.) for 2 d	Lycopene (4 and 6 mg/kg) for 7 d after ischemia	Reducing disorder of the cell morphology and apoptosis via ↑SOD, CAT, GSH and GPx, ↓IL-6,1*β*, TNF-*α*, Bax/Bcl-2, JNK/(ERK, STAT1,3), Cyt-c, and caspase-3	[98]
Sprague-Dawley rats	ISP (100 mg/kg, i.p.) at 7 and 8 d	Lycopene 3 mg/kg and quercetin 80 mg/kg p.o. for 1-10 d	Reducing cardiotoxicity (↓CK-MB, LDH, MYO, and TROP) and oxidative stress (↓MDA) via ↑GST*μ*, SOD1, SOD2, CAT, and Bcl-2	[102]

**Table 2:** Effects of lycopene on myocardial I/R injury.

Species	I/R Models	Treatments	Effects and mechanisms	Ref
Wistar rats	I/R (30 min/120 min)	Lycopene (40 mg/kg/d, i.p.) for 5 d before I/R	Reducing myocardial infarction area, apoptosis, and the opening of mPTP via ↓APAF-1, caspase-3, caspase-9, Bax, Bax/Bcl-2, Cyt-c, and ↑Bcl-2	[120]
Wistar rats	I/R (30 min/60 min) with HCD	Lycopene (20 mg/kg/d, i.p.) for 5 d before I/R	Decreasing myocardial infarction area and apoptosis and improving cardiac functions via ↓GRP78 and CHOP, Cyt-c, caspase-3, caspase-9, p-GSK-3*β* and ↑p-Akt, and p-ERK1/2	[113]
C57BL/6 mouse; HL-1cell	LAD I/R (20 min/4 h); H/R (2 h/2 h)	Lycopene (33.5 mg/kg∗ b.w., i.v.) after ischemia; lycopene (1, 2, and 4 *μ*M) after hypoxia	Reducing myocardial infarction area and apoptosis via inhibiting the level of p-JNK and ROS and the activation ERK1/2	[107]
Sprague-Dawley rats	Left coronary artery I/R (30 min/24 h)	Lycopene (1.5 mg/kg/d, i.g.) for 30 d before I/R	Reducing myocardial infarction area, apoptosis, and mtDNA damage via ↓MDA, Cyt-c, caspase-3, ROS level, and ↑Tfam	[121]
Wistar rats	LAD I/R (45 min/1 h)	Lycopene (1 mg/kg, i.g.) for 31 d before I/R	Reducing oxidative stress and disorder of the cell morphology via ↑GSH and GSH-Px level and ↓MDA	[122]
Sprague-Dawley rats	I/R (30 min/2 h)	Lycopene (0.088 mg/d, i.g.) for 3 w before I/R	Improving ventricular functions, reducing myocardial infarction area and apoptosis via ↓MDA and ROS	[123]
H9C2 cardiomyocytes	H/R (4 h/4 h)	Lycopene (10 *μ*M) for 4 h before H/R	Reducing ER stress and apoptosis via inhibiting GRP78, CHOP, caspase-12, p-JNK, and LDH	[124]
H9C2 cardiomyocytes	H/R (16 h/2 h)	Lycopene (2.5 *μ*M) for 48 h before H/R	Increasing autophagy and reducing apoptosis via ↓Bax/Bcl-2, caspase-3, ↑Beclin 1, MAP1LC3B, and p-AMPK	[125]
C57BL/6 mouse cardiomyocytes	H/R (4 h/6 h)	Lycopene (5 *μ*M) for 4 h before H/R	Reducing ER stress and apoptosis via ↑AMPK, ↓caspase-3, caspase-12, CHOP, Bax/Bcl-2, sXbp-1, eIF2*α*, GRP78, ROS, and ATF6	[126]
C57BL/6 mouse cardiomyocytes	H/R (4 h/8 h)	Lycopene (5 *μ*M) for 4 h before H/R	Reducing apoptosis via inhibiting mPTP opening and decreasing caspase-3 and Cyt-c	[127]

**Table 3:** Effects of lycopene on hepatic I/R injury.

Species	I/R Models	Treatments	Effects and mechanisms	Ref
Sprague-Dawley rats	The portal vein, the bile duct, and the hepatic artery I/R (45 min/60 min)	Lycopene (2.5 and 5 mg/kg, i.p.) at 60 min before I/R	Reducing liver damage via ↑CAT and ↓ROS, ALY, AST, LDH, and MDA	[136]
C57BL/6 mice	The portal vein and hepatic artery I/R (90 min/6 h)	Lycopene (20 mg/kg, i.g.) for 2 weeks before I/R	Inhibiting NLRP3 inflammasome and apoptosis and enhancing autophagy via ↑Bcl-2, LC3B, IL-10, ↓TNF-*α*, IL-6, MPO, IL-1*β*, caspase-1, caspase-3, and p62 and activating Nrf2/HO-1	[137]
AML12 hepatic cells	H/R (12 h/4 h)	Lycopene (10 *μ*M) for 4 h before H/R	Reducing apoptosis and oxidative stress via ↓TNF-*α*, IL-6, MDA, and ROS and activating Nrf2/HO-1	[138]
Wistar rats	H/R (45 min/2 h)	Lycopene (25 mg/kg/d, p.o.) for 14 d before H/R	Relieving edema and reducing oxidative stress via reducing ALT, AST, LDH, and MDA	[139]

**Table 4:** Effects of lycopene on cerebral I/R injury.

Species	I/R models	Treatments	Effects and mechanisms	Ref
Mongolian gerbils	Bilateral common carotid artery I/R (10 min/3 h)	Lycopene (5 mg/100 g, in diet) after ischemia	Inhibiting apoptosis via increasing Bcl-2 and SOD, reducing caspase-3 and Bax	[165]
C57BL/6 mice	Bilateral common carotid artery I/R (20 min/24 h)	Lycopene (20 mg/kg, i.p.) for 7 d before I/R	Improving neurological function scores, reducing oxidative stress and apoptosis via reducing ROS, and increasing GSH and Nrf2/HO-1	[166]
Sprague-Dawley rats	Middle cerebral artery I/R (2 h/24 h)	Lycopene (5 and 20 mg, i.g.) for 15 d before I/R	Reducing infarct area and nerve function defect via ↑SOD, CAT, Bcl-2, HIF-1*α*, ↓iNOS, and MDA	[167]
Sprague-Dawley rats	Middle cerebral artery I/R (60 min/7 d)	L-LYC (6 mg/d, i.g.) for 14 d before I/R	Reducing infarct area and nerve function defect via reducing iron content (↓hepcidin and L-ferritin and ↑FPN1), oxidation (↑SOD, CAT, GSH, ↓ROS, iNOS, and NO), apoptosis (↑Bcl-2, ↓caspase-3, and JNK/MAPK), and inflammation (↓IL-6 and p-STAT3)	[159]
Wistar rats	Middle cerebral artery I/R (1 h/24 h)	Lycopene (4 mg/kg, i.v.) twice at 15 min before ischemia and reperfusion	Reducing infarct area via inhibiting microglia activation, the production of NO, and lipid peroxidation and scavenging ROS	[168]

## Abbreviations

ACC: Acetyl-CoA carboxylase
Akt: Protein kinase B
ALT: Alanine aminotransferase
AMPK: Adenosine monophosphate-activated protein kinase
APAF-1: Apoptotic peptidase activating factor 1
ARE: Antioxidant response element
AST: Aspartate aminotransferase
ATF6: Activating transcription factor 6
ATM: Ataxia telangiectasia mutated protein
Bax: Bcl-2-associated X protein
Bcl-2: B-cell lymphoma 2
BT: Bacterial translocation
CAT: Catalase
CHOP: C/EBP homologous protein
CK-MB: Creatine kinase isoenzymes
COX: Cyclooxygenase
CRP: C-reactive protein
Cyt-c: Cytochrome c
eIF2*α*: Eukaryotic translation initiation factor 2 subunit-*α*
ER: Endoplasmic reticulum
ERK: Extracellular regulated protein kinases
F2IsoP: F2-Isoprostane
GRP78: Glucose-regulated proteins 78
GSH: Glutathione
GSH-Px: Glutathione peroxidase
GSK-3*β*: Glycogen synthase kinase-3*β*
GST: Glutathione S-transferase
HCD: High-cholesterol diet
Hes-1: Hairy enhancer of split
HIF-1: Hypoxia inducible factor-1
HO-1: Heme oxygenase 1
hs-CRP: Hypersensitive C-reactive protein
IGF: Insulin-like growth factors
IL: Interleukin
JAK: The Janus kinase
JNK: c-Jun N-terminal kinase
KEAP1: Kelch-like erythroid cell-derived protein with CNC homology-associated protein 1
LAD: Left anterior descending
LDL: Low-density lipoprotein
LDH: Lactate dehydrogenase
LV: Left ventricular
MAP1LC3B: Microtubule-associated protein 1-light chain 3*β*
MDA: Malonic dialdehyde
MMP-9: Matrix metalloproteinase-9
MPO: Myeloperoxidase
mPTP: Mitochondrial permeability transition pore
mtDNA: Mitochondrial DNA
mTOR: Mammalian target of rapamycin
MYO: Myoglobin
NF-*κ*B: Nuclear factor-kappa B
NLRP3: NOD-like receptor protein 3
NOS: Nitric oxide synthase
NQO-1: NADPH quinine oxidoreductase-1
Nrf2: Nuclear factor-erythroid 2-related factor 2
PCI: Percutaneous coronary intervention
PI3K: Phosphoinositide 3-kinase
PKC: Protein kinase C
ROS: Reactive oxygen species
SIRT-1: Sirtuin 1
sIgA: Secretory immunoglobulin A
SOD: Superoxide dismutase
STAT: Signal transducer and activator of transcription
sXbp-: Splices X-box binding proteins-1
Tfam: Mitochondrial transcription factor A
TLR-2: Toll-like receptor 2
TNF: Tumor necrosis factor
TnI: Troponin I
TROP: Troponin T.
